# Analysis of the Effect of the COVID-19 Pandemic on Syphilis in Susceptible Populations: Men Who Have Sex with Men, People Living with HIV, and Patients with Gestational and Congenital Syphilis—A Narrative Review

**DOI:** 10.3390/microorganisms13061205

**Published:** 2025-05-25

**Authors:** Natalia Welc, Wiktoria Frącz, Rafał Olejniczak, Ryszard Żaba, Kevin Kavanagh

**Affiliations:** 1 Department of Dermatology and Venereology, Poznan University of Medical Sciences, 61701 Poznan, Poland; dermatologia@spsk2.pl; 2 Department of Physiotherapy, Poznan University of Medical Sciences, 61701 Poznan, Poland; 3 Faculty of Health Sciences, University of Zaragoza, 50009 Zaragoza, Spain; 4 Department of Urology, Collegium Medicum, University of Zielona Gora, 65417 Zielona Gora, Poland; 5 Department of Biology, Maynooth University, W23 F2K8 Maynooth, Ireland; biology.department@mu.ie

**Keywords:** syphilis, COVID-19, pandemic, congenital syphilis, gestational syphilis, men who have sex with men, MSM, people living with HIV, PLWH, HIV, blood donors

## Abstract

The COVID-19 pandemic triggered a public health crisis that significantly impacted sexually transmitted infections (STIs), particularly syphilis. However, data on syphilis incidence during the pandemic remains inconsistent globally. Key groups affected include women of reproductive age, pregnant women, individuals living with HIV, and men who have sex with men (MSM). This paper reviews available literature from databases such as PubMed, Scopus, and Google Scholar to analyse the pandemic’s influence on congenital and gestational syphilis, focusing on high-risk populations. We discuss the pandemic’s impact on the incidence of gestational and congenital syphilis, including changes in screening and treatment protocols. Additionally, we examine alterations in syphilis prevalence and testing among people living with HIV and MSM, including implications observed in blood donors. The findings underscore the consequences of impaired STI diagnostics for public health. We emphasise the need for uninterrupted access to diagnostics and treatment during public health crises. To prevent rising syphilis rates post-pandemic, it is crucial to implement robust education and accessible testing measures.

## 1. Introduction

The COVID-19 pandemic, which began in China at the end of 2019, resulted in a global public health crisis. Infection with SARS-CoV-2 was characterised by pneumonia and a systemic inflammatory response, leading to a cytokine storm. The fatality rate eventually reached approximately 3% [1]. To mitigate the spread of the coronavirus, governments implemented numerous measures, such as closing schools, universities, and various public places and, when possible, limiting their capacity. The lockdown included home confinement, curfews, and border closures [2]. As these preventive actions aimed to reduce human contact, it was assumed that the prevalence of sexually transmitted infections (STIs), including syphilis, would decrease. In many locations, like Hungary, Greece, Japan, or New York City, USA, a decline in syphilis cases was observed; however, it remains unclear whether this was genuine or if diagnostics and reporting fell short [3,4,5,6]. The influx of COVID-19 patients often limited in-person care for others. Consequently, routine and screening appointments were less frequent, along with a reduction in non-emergency visits [3]. In several facilities, decreases in STI testing were observed, especially in the early phases of the COVID-19 pandemic, which could have led to underdiagnosing and underreporting of STIs during this particular period [7,8]. Conversely, as several studies have already indicated, syphilis rates were not lower in all areas, such as in California, USA, or Belgrade, Serbia and Messina, Italy in Europe [9,10,11].

Given that STIs present a growing public health concern, neglecting asymptomatic and undiagnosed individuals during the COVID-19 pandemic could have delayed diagnoses and led to more severe complications. Attention should be directed toward specific subgroups of the population, such as pregnant women or people living with HIV (PLWH), as the consequences of the infection pose a significant risk to these patients’ health and lives. Some population groups are more severely impacted by syphilis, including men who have sex with men (MSM), transgender individuals, and people living with HIV (PLWH) [12]. Since syphilis and HIV are both sexually transmitted, co-infection often occurs. Infection with one may enhance the chance of acquiring and spreading the other; therefore, individuals living with HIV should receive particular attention during screening [13]. Additionally, blood safety is a vital component of global healthcare systems, characterised by strict screening, testing, and processing protocols that aim to reduce the risk of transfusion-transmitted infections (TTIs), so variability in STI incidence can be observed through the screening of blood donors [14].

Syphilis, a sexually transmitted infection (STI) caused by the *Treponema pallidum* bacterium, represents a major global health crisis that particularly impacts high-risk demographics, including men who have sex with men. The disease spreads primarily through direct skin contact during sexual intercourse with someone who has active primary or secondary lesions. Additional transmission routes include oral sex, transmission from mother to child during pregnancy (leading to congenital syphilis), and exposure to infected blood during transfusions [15].

Infected individuals typically move through various stages of the disease—primary, secondary, latent, and tertiary—over at least a decade. Early syphilis includes infections transmitted through sexual contact, covering primary, secondary, and early latent stages. A person with primary syphilis typically presents a painful ulcer (chancre) or multiple lesions in the genital area, along with swollen lymph nodes, about three weeks post-infection. These lesions often heal spontaneously. Secondary symptoms may develop 6 to 8 weeks later, such as fever, headaches, and a characteristic rash on the flanks, shoulders, arms, chest, or back, often affecting the palms and soles. As these symptoms fade, patients enter a latent phase that can last for years. If untreated, individuals risk progressing to tertiary syphilis, leading to serious complications like cardiac or neurological disorders, gummas, or bone issues [16,17].

Syphilis is a significant public health challenge, exacerbated by the pandemic’s impact on healthcare. Cases have risen, becoming a health threat to millions. According to the European Centre for Disease Prevention and Control (ECDC), in countries under ECDC surveillance from 2013 to 2022, notification rates of syphilis infections per 100,000 population steadily increased until 2019, decreased in 2020 due to COVID-19, then surged again in 2021, ultimately hitting record highs in 2022 and 2023 (8.8 and 9.9 cases per 100,000, respectively) [18]. Reports of congenital syphilis from 2021 to 2023 indicate increased notifications within the EU/EEA, reversing the 2020 decline, with a rate of 2.7 per 100,000 live births in 2023 [19]. In the USA, the Centres for Disease Control and Prevention reported a concerning congenital syphilis rate of 105.8 cases per 100,000 live births, steadily rising since 2019 (50.3, 59.9, 78.6, and 102.8 per 100,000 in 2019, 2020, 2021, and 2022, respectively) [20]. In 2023, there were 209,253 reported syphilis cases at all stages, the highest since 1950, representing a 1.0% increase from 2022. While overall cases rose by 1.0% from 2022, the incidence rate per 100,000 remained stable, fluctuating less than 1% (from 61.1 to 61.3 per 100,000). However, trends varied across the different stages of syphilis [21]. These data highlight the urgent need for syphilis prevention and treatment.

Penicillin is the primary drug for treating syphilis. The choice of penicillin preparation, dosage, delivery method, and treatment duration depends on the syphilis stage and clinical presentation. Selecting the correct penicillin formulation is critical, as *T. pallidum* may exist in isolated areas like the central nervous system, making it challenging to achieve adequate penicillin levels. Although several macrolide or tetracycline-resistant *T. pallidum* strains were observed, fortunately, no cases of penicillin resistance have been reported. Thus, penicillin remains the preferred treatment, with doxycycline, tetracycline, ceftriaxone, and amoxicillin considered acceptable alternatives [22,23].

In this narrative review, we present available data on the influence of the COVID-19 pandemic on the prevalence of gestational and congenital syphilis, as well as among populations of PLWH (people living with HIV) and men who have sex with men (MSM). Furthermore, we report the findings from blood donors, discuss difficulties in diagnosing STIs during the pandemic and highlight some unusual cases of syphilis associated with COVID-19 infection or vaccination.

## 2. Materials and Methods

The review was prepared using available literature from the PubMed, Scopus, and Google Scholar databases by searching keywords such as “COVID-19”, “syphilis”, “congenital syphilis”, “MSM”, “blood”, “HIV” and “PLWH”. Three researchers individually reviewed the databases on 1 February 2025. Grey literature was not included in the review. Only articles connected directly to the subject of the impact of the COVID-19 pandemic and congenital syphilis, gestational syphilis, syphilis in MSM, syphilis in PLWH, and syphilis in blood donors were selected. Twenty-seven papers from three online databases were included in the review, of which seven were on the influence of the COVID-19 pandemic on gestational and congenital syphilis, eight on PLWH and MSM groups, and twelve on blood donors and atypical cases with diagnostic issues.

## 3. Discussion

### 3.1. Congenital and Gestational Syphilis

Congenital syphilis (CS) is a result of an intrauterine infection with *T. pallidum* in a fetus. It can occur in any stage of pregnancy and is frequently associated with an increased risk of preterm birth and perinatal death [24]. Some of the signs of syphilis infection in utero can be revealed in the ultrasound examination after 18 weeks of pregnancy, including hepatomegaly, foetal anaemia, placentomegaly, ascites or foetal hydrops [25]. CS is divided into early and late CS, where the differentiating factor is the age of the child; a patient is diagnosed with early syphilis under two years of age, and a patient over two years of age is classified with late syphilis [26]. Early CS symptoms involve rhinitis, “snuffles”, rash, jaundice, hepatosplenomegaly, and fever. Late CS is marked by rhagades (fissures at the mouth’s corners), saddle nose (loss of nasal height from hard palate defect), and Hutchinson’s triad (blunted upper incisors, interstitial keratitis, and eighth cranial nerve deafness) [27]. The gestational screening tests combine non-treponemal (RPR-Rapid Plasma Reagin, and VDRL-Venereal Disease Research Laboratory) and treponemal (TPHA-Treponema Pallidum Hemagglutination Assay, FTA-Fluorescent Treponemal Antibody, and FTA-ABS-Fluorescent Treponemal Antibody Absorption) tests [28]. The effective treatment for syphilis during pregnancy remains benzathine penicillin, according to the Centres for Disease Control and Prevention (CDC) [29]. Recent medical advancements have made CS uncommon due to effective screening, detection, and antibiotic prevention strategies. Nevertheless, there has been a significant increase in adult and maternal syphilis cases in developed nations in recent years [27]. Following the risk for public health, a global target for the elimination of congenital syphilis of ≤50 cases of congenital syphilis per 100,000 live births has been set by the World Health Organization (WHO) to be achieved by 2030 in 80% of countries [30].

There is limited data on the influence of the COVID-19 pandemic on CS and syphilis in pregnant women or the subgroup of women of reproductive age. In the systematic review from 2023, Pascoal et al. concluded that the absence or late onset of prenatal care was associated with the risk of CS, which correlates with the available research [31]. The report from Chicago observed a 74% rise in CS cases in 2020–2022 compared to the pre-pandemic period (2015–2019). Almost half of them (48.3%) were declared as due to inadequate maternal treatment despite timely syphilis diagnoses. The authors mention the late identification of seroconversion during pregnancy, which was most notably visible in 2020 as a result of the decrease in testing due to the closure of STI clinics and a preference for telemedicine instead of in-person prenatal care. Moreover, a rise in the primary or secondary syphilis cases in females of reproductive age during the pandemic was assessed at 22% [32]. A surge in CS care rates during the pandemic was also observed in the US Military Health System, representing a fully insured population. Teng et al. discovered that the rates of care for CS in this insured group were significantly lower than the national averages. Nevertheless, they saw a notable elevation during the COVID-19 pandemic. The care rates were higher in children of lower-ranked military members. The study revealed that healthcare disruptions during the pandemic affected all patients, regardless of income or insurance coverage [33]. In Saint Laurent du Maroni in French Guiana, there was a sharp growth in the number of CS cases during 2020–2021 in the COVID-19 pandemic–the incidence was almost twice as high as the global estimates, probably due to disturbances in prenatal care and lack of syphilis screening [34]. Furthermore, the researchers from New Jersey University Hospital found that even though throughout the pre-COVID period (2016–2019) syphilis rates among mothers and infants remained relatively stable, there was a notable increase at the onset of the COVID-19 pandemic, which continued thereafter. Interestingly, during 2020 and 2021, the rates of CS were significantly higher than both state and national levels. Additionally, infants born to mothers with a history of opioid use, those experiencing homelessness, or those who were employed at the time of delivery were markedly more likely to develop CS. This latter trend may be attributed to better access to healthcare for employed patients. Moreover, the authors noted an increase in the vertical transmission rate, which rose in 2020 after declining to 33.3% in 2019. This suggests not only a consequence of higher maternal infections but also reflects ineffective prevention methods for vertical transmission during the pandemic [35].

On the other hand, Pinheiro et al. studied the influence of the COVID-19 pandemic on congenital and gestational syphilis in Brazil and found a reduction of 9% and 1%, respectively. The slight reduction entails both lockdown consequences as well as the ever-growing prevalence of syphilis in Brazil since 2010 [36]. Furthermore, according to a study from the state of Paraná, Brazil, there was a substantial general increase in the detection rates of congenital and gestational syphilis throughout 2007–2021, regardless of maternal age. The decline observed in this study during the COVID-19 pandemic highlights the need to strengthen health education programmes, expand testing and treatment alternatives for expectant mothers and their partners, and improve access to adequate maternal and child healthcare [37]. Another study from Brazil supports these findings, as the authors observed that antenatal care was more effective after the COVID-19 pandemic than during it. Patients reported difficulty scheduling appointments or exams and complained about reduced healthcare staff. It was noted that when antenatal care met the goal criteria, these patients were twice as likely to have been thoroughly investigated for syphilis, which is essential for appropriate treatment [38]. The summary of the most important findings on the impact of the COVID-19 pandemic on congenital and gestational syphilis is presented in Table 1.

### 3.2. Syphilis in People Living with HIV and Men Who Have Sex with Men

The impact of the COVID-19 pandemic on the incidence of syphilis in PLWH and MSM has been significant. The importance of findings within these groups must be emphasised. In Lebanon, the proportion of MSM among those who tested positive for any STI remained unchanged before and after the COVID-19 pandemic, underlining a persistent issue [39]. According to Lee et al., there was a decline in newly diagnosed HIV cases in Korea during 2020–2021; however, an increase was already observable in 2022. From 2017 to 2019, syphilis rates among PLWH increased gradually, rising from 1.85 cases per 100,000 population in 2014–2016 to 3.0 cases per 100,000 in 2017–2019. This trend intensified during the COVID-19 pandemic (2020–2022), peaking at 3.3 cases per 100,000 population, which may reflect similar patterns observed in the general population of Korea. The authors found that men experiencing new syphilis cases during the pandemic were more likely to be younger, identify as MSM, have a history of syphilis infections, and have been previously incarcerated, suggesting potential target populations for testing and sexual health education within the PLWH community [40]. A study from Croatia analysing the influence of the COVID-19 pandemic on syphilis incidence among males living with HIV (MLWH) observed an increase of 91.4% in 2020 compared to 2019. Higher syphilis rates were noted among MSM, MLWH with a baseline history of syphilis, and MLWH with a more recent HIV diagnosis. However, males with clinical AIDS were characterised by a lower syphilis rate. The authors emphasise that the escalation in syphilis cases occurred during a period of slightly higher syphilis testing rates [41]. Another study from Thessaloniki, Greece, regarding syphilis among PLWH at elevated risk for STIs during 2019–2022 also indicated higher rates of syphilis in MSM and younger patients, with rates continuing to rise into 2022. The researchers observed that the shift in perception of HIV infection as a manageable chronic illness has unintentionally led to a diminished emphasis on safe sex practices among certain groups, contributing to an increase in new HIV diagnoses and co-infections with syphilis, a trend likely unchanged during the COVID-19 pandemic [12]. Conversely, research conducted at Liège University Hospital suggests that significantly fewer PLWH underwent screening in 2020 compared to 2019, implying that the increases identified in the aforementioned studies in some cases may be underestimated [42]. Access to healthcare services was crucial in continuously diagnosing and treating STIs. In Ireland, Shanley et al. observed that gay, bisexual, and other men who have sex with men (GBMSM) aged over 56, not previously tested for HIV and with a medium or lower level of education were at the highest risk of not receiving proper care. Contrarily, GBMSM already diagnosed with an HIV infection had the highest odds of successful medical attention [43].

Interviewing some GBMSM in Indiana, USA, revealed that some people sought mitigating strategies to avoid a COVID-19 infection; however, the feeling of an inevitable infection led to a decrease in preventive measures [44]. The survey conducted among MSM in Columbus, OH, USA, revealed that during the COVID-19 pandemic, sexual frequency varied over several months. From April to July 2020, nearly three-quarters reported being less inclined to seek new partners. Despite initial declines in sexual activity, rates rose from August to December 2020, possibly due to what is termed “pandemic fatigue”, and remained stable from January until May 2021. Condom use did not increase, likely due to the focus on respiratory transmission. The survey indicated that men of colour reported higher sexual frequency overall. Interestingly, as in-person sexual encounters declined, many individuals turned to online sexual activity [45].

All things considered, STI outpatient care, especially for PLWH and other key populations, should remain unaffected even in times of a national health crisis like the pandemic. The summary of the most important findings on the sexually transmitted infection-related problems and syphilis incidence in PLWH and MSM during the COVID-19 pandemic is presented in Table 2.

### 3.3. Syphilis in Blood Donors

Blood donors are routinely tested for communicable diseases, including STIs and syphilis, leading to valuable insights regarding the impact of the COVID-19 pandemic on this specific group. The tertiary care centre recorded a significant increase in syphilis prevalence among blood donors in both 2020 and 2021, while blood donations fell by 53.79% and 34.4% in those years, respectively [46]. Interestingly, in Shenzhen, the number of blood donors rose in 2020. However, the blood was more frequently donated by individuals with higher education levels, as well as by local and repeat blood donors, compared to previous years. During the COVID-19 pandemic, syphilis prevalence was notably lower among female and repeat blood donors than in 2019, yet it increased among first-time blood donors [47]. A significant rise in syphilis infections during the pandemic was also observed in blood donors from Punjab, India [48].

A retrospective analysis of over 65,000 blood donors between 2018 and 2022 from a hospital in Thailand revealed that the COVID-19 pandemic increased syphilis prevalence in this group by 9.2%, noting a rise of 22% in males and a decrease of 5% in females. During this period, syphilis was more frequently detected in younger individuals (ages 17–40) and older adults (ages over 51). In contrast, there was a significant fall in syphilis prevalence among those aged 41–50. Furthermore, the rate of syphilis in first-time donors nearly doubled, while a 9% decline was observed in repeat donors. The rationale behind these findings is unclear [14].

In the article from Maryland, USA, Miller et al. [49] noted an increase in unconfirmed syphilis reactivity rates from 2018 to 2023 despite a decrease in blood donations during these years. The authors imply that viral infections such as COVID-19 or vaccinations may lead to false positive results for certain infectious diseases, such as syphilis, particularly as there was an autumnal peak in positive reactivity rates [49]. This theory is substantiated by a study of sera from thirty-eight participants. RPR tests revealed false reactivity, with baseline nonreactive samples from seven patients becoming reactive after vaccination. All cases were associated with the Moderna vaccine. The authors recommend that serological tests yielding inconsistent results with the clinical presentation following COVID-19 vaccination should be retested. Specific assays could have effects lasting over five months. The RPR test is particularly susceptible to false positives, and assay results can vary significantly [50]. Researchers from Hennepin County Medical Center in Minneapolis, Minnesota, USA, also found an increase in false-positive RPR rates in the screening (5% to 8.6%) and pregnant groups (4.4% to 7.5%) from 2019 to 2021 when using the BioPlex2200 RPR assay (BioRad Laboratories, Hercules, CA, USA). The authors attribute this finding to the improved analytical sensitivity of this assay for antilipoidal antibodies compared to manual RPR methods, which may result in higher susceptibility to false positives post-vaccination. It was also observed that over 90% of RPR false positives had titers less than 1:4 [51]. A case of a post-COVID-19 false positive for VDRL was also reported [52]. Conversely, two cases of unmasking secondary syphilis after COVID-19 vaccination were documented, both occurring approximately two weeks post-injection [53,54]. An unusual case of secondary syphilis mimicking erythema multiforme in an HIV-positive patient was observed in Indonesia, which emphasises numerous potential dermatological manifestations of this disease [55]. Furthermore, a case of a male with concurrent COVID-19 infection and primary syphilis was reported, which likely represents one of many patients who could acquire both infections simultaneously [56].

### 3.4. The Issue of Impaired Diagnostics of STIs During the COVID-19 Pandemic

The impact of the COVID-19 pandemic on diagnosing and treating STIs is undeniable. Still, some challenges associated with transferring health services to those infected with coronavirus were overlooked, and several changes should be implemented.

An interesting observation from Brazil reveals that a mobility reduction exceeding 50% resulted in fewer men being tested, alongside a lower median number of syphilis tests conducted weekly and a higher percentage of positive syphilis results. This indicates a considerable impact of lockdowns on using available diagnostic options for STIs in the general population, which may also apply to key populations [57]. Additionally, there were declines in STI testing, including for syphilis, among younger populations in England. Testing frequency for those aged 15 to 19 dropped by 42%, while for those aged 20 to 24, it decreased by 33% when comparing 2020 to 2019. The number of diagnostic tests experienced the most significant declines among heterosexual men (46%), heterosexual women (34%), and MSM (20%) [58]. These reports highlight the need for uninterrupted access to such services to ensure proper diagnostic and therapeutic processes.

One possible approach to improving sexual health is to incorporate at-home STI testing into online healthcare services, leveraging the growth in telemedicine, which was driven directly by the COVID-19 pandemic [59]. However, the shift to telemedicine may create additional challenges for disadvantaged individuals who lack the necessary technology and literacy for this new approach [10]. Moreover, recent advancements in STI detection technology led to the development of point-of-care molecular tests for chlamydiasis, gonorrhoea, trichomoniasis, and syphilis serology. Over-the-counter tests will soon enable self-testing without a healthcare provider [60]. As reported by Towns et al. [61], syphilis self-testing (SST) could benefit significant demographics, particularly MSM. It engages more individuals than facility-based testing, is straightforward to use, provides prompt results, and saves time and money. Additionally, the adoption of SST was 88% among MSM, with few reports of social harm reflecting HIV self-testing experiences. Nevertheless, a key limitation of the available SST kits is their capacity to detect only treponemal antibodies, which fail to differentiate between past and current syphilis infections, thereby restricting their applicability to those without a history of syphilis. This presents challenges for high-incidence groups, such as MSM and transgender women. A combined treponemal and non-treponemal rapid test could improve self-testing options, particularly for populations with a high disease prevalence [61]. Although SST appears to be an essential and beneficial tool for STI diagnostics, one cannot overlook its limitations regarding STI counselling, which may lead to reduced partner tracing, incidence reporting, or even hinder access to appropriate care. These challenges should be discussed and addressed before implementing such options in the open market.

Following the COVID-19 pandemic, recommendations for future health crises have surfaced. Challenges to address include a shortage of personal protective equipment, insufficient staff, limited resources, laboratory services, overcrowded waiting rooms, and issues with infection control. Directors and staff of STI clinics must stay informed about the latest protocols and standing orders for delivering clinical services. These strategies are essential to ensure continued access to STI services during the global COVID-19 pandemic [62].

## 4. Conclusions

The global COVID-19 pandemic has imparted valuable lessons to the medical community. Governments must ensure that STI prevention and healthcare services for key populations remain accessible and adaptable to meet the patients’ needs during these unprecedented times. Failing to do so could result in serious repercussions related to communicable diseases. Comprehensive sexual education and ongoing testing that facilitate timely diagnosis and treatment are essential for addressing future public health crises, including pandemics.

## Figures and Tables

**Table 1:** Congenital (CS) and gestational syphilis (GS) during the COVID-19 pandemic.

Authors	Location	Period Analysed	Observations
Cejtin et al. [32]	Chicago, IL, USA	2015–2019 vs. 2020–2022	During 2020–2022, syphilis cases in females of reproductive age increased an average of 22.1% per year and CS cases an average of 74.1% per year.
Teng et al. [33]	Military Health System database, USA	Pre-COVID-19 (March 2018 to February 2020), pandemic year 1 (March 2020 to February 2021), pandemic year 2 (March 2021 to February 2022)	Compared to pre-COVID-19, CS care rates increased in pandemic years 1 and 2 (adjusted rate ratio 2.76 and 5.52, respectively).
Nacher et al. [34]	Saint Laurent du Maroni, French Guiana	2020–2021	In 2021, CS was observed in 808 per 100,000 live births (27 cases for 3340 deliveries), nearly two times more than global estimates.
Heiman et al. [35]	New Jersey University Hospital, Newark, NJ, USA	1 January 2016–1 June 2022	GS and CS rates increased over the 5 years (*p* < 0.001), particularly when comparing pre- and post-COVID-19 (*p* < 0.001).
Pinheiro et al. [36]	Brazil (Brazilian Notifiable Diseases Information System database)	2019–2020	GS rates declined by 1.1% and CS rates by 9.2% between 2019 and 2020.
de Oliveira et al. [37]	Paraná state, Brazil	2007–2021	GS and CS detection rates increased in 2007–2021, with a decrease during the pandemic. GS rates were rising in 2007–2016 (annual percentage change, APC, 41.4%) and 2017–2019 (APC 13.3%), and declining in 2019–2021 (APC−30.9%), similarly for CS (2007–2015: APC 32.8%, 2016–2019: APC 11.5%, 2019–2021: APC−32.4%).
Beatrici et al. [38]	University Hospital of Florianopolis, Florianópolis, Brazil	2020–2022	Insufficient antenatal care was notably related to pregnancies in 2020. Receiving proper prenatal care was associated with a two-fold increase in the chances of being tested for syphilis.

**Table 2:** Sexually transmitted infection-related problems and syphilis incidence in PLWH and MSM during the COVID-19 pandemic.

Authors	Location	Period Analysed	Group	Observations
Nanoudis et al. [12]	Thessaloniki, Greece	January 2019–December 2022	PLWH	The incidence of syphilis decreased in 2020 compared to 2019, then showed a steady increase until 2022. 90.5% of patients were MSM.
Sunji et al. [39]	Beirut, Lebanon	March 2019–February 2020 vs. March 2020–February 2021	MSM	The MSM share was higher after the onset of the pandemic than before (39.8% vs. 35.2%; *p* = 0.043). The MSM proportion among those who tested positive for any STI was unchanged between the pre- and post-COVID-19 periods.
Lee et al. [40]	Republic of Korea	2005–2022	PLWH	Syphilis incidence in PLWH increased to 3.33 in 2020–2022 (the COVID-19 pandemic). Young age, MSM, and a history of incarceration were increasing the risk of acquiring syphilis.
Begovac et al. [41]	Croatia	2018–2021	Male persons living with HIV (MLWH)	The occurrence of syphilis rose from 2018 to 2021, with a notable spike between 2019 and 2020 (91.4%). The syphilis testing rate was the highest in 2021.
El Moussaoui et al. [42]	Liège, Belgium	2019–2020	PLWH	When comparing 2020 to 2019, there was a decrease in the number of new HIV diagnoses, in the number of consultations at the HIV clinic, in the number of viral load assays and blood CD4+ T-cells count analyses performed, and in the frequency of screening of PLWH for hepatitis C and syphilis.
Shanley et al. [43]	Ireland	2021	Gay, bisexual and other men who have sex with men (GBMSM)	GBMSM aged over 56, not previously tested for HIV and with medium and low education had the lowest odds of successfully accessing services. GBMSM with HIV were most likely to be able to access services successfully.
Loosier et al. [44]	Marion County, IN, USA	2020–2021	GBMSM	Some GBMSM adopted strategies to minimise COVID-19 risk while connecting with partners, while others felt that acquiring the virus was inevitable, leading to less focus on preventive measures during sexual activities.
Ricks et al. [45]	Columbus, Ohio; Baltimore, Maryland; and Chicago, IL, USA	April 2020- May 2021	MSM	At the beginning of the pandemic (April–July 2020), the majority of men (67%) continued to engage in sex, and the number was increasing over time (79% in August–December 2020 and 77% in January–May 2021).

## Data Availability

No new data were created or analyzed in this study.

## Abbreviations

The following abbreviations are used in this manuscript:

STI	Sexually Transmitted Infection
HIV	Human Immunodeficiency Virus
AIDS	Acquired Immune Deficiency Syndrome
MSM	Men who have Sex with Men
PLWH	People Living With HIV
MLWH	Men Living With HIV
GBMSM	Gay, Bisexual and other Men who have Sex with Men
CS	Congenital Syphilis
